# In Utero Caffeine Exposure Induces Transgenerational Effects on the Adult Heart

**DOI:** 10.1038/srep34106

**Published:** 2016-09-28

**Authors:** Xiefan Fang, Ryan R. Poulsen, Scott A. Rivkees, Christopher C. Wendler

**Affiliations:** 1 Child Health Research Institute, Department of Pediatrics, College of Medicine, University of Florida, Gainesville, FL 32610, USA.

## Abstract

Each year millions of pregnant woman are exposed to caffeine, which acts to antagonize adenosine action. The long-term consequences of this exposure on the developing fetus are largely unknown, although in animal models we have found adverse effects on cardiac function. To assess if these effects are transmitted transgenerationally, we exposed pregnant mice to caffeine equivalent to 2–4 cups of coffee at two embryonic stages. Embryos (F1 generation) exposed to caffeine early from embryonic (E) day 6.5–9.5 developed a phenotype similar to dilated cardiomyopathy by 1 year of age. Embryos exposed to caffeine later (E10.5–13.5) were not affected. We next examined the F2 generation and F3 generation of mice exposed to caffeine from E10.5–13.5, as this coincides with germ cell development. These F2 generation adult mice developed a cardiac phenotype similar to hypertrophic cardiomyopathy. The F3 generation exhibited morphological changes in adult hearts, including increased mass. This report shows that *in utero* caffeine exposure has long-term effects into adulthood and that prenatal caffeine exposure can exert adverse transgenerational effects on adult cardiac function.

It is well recognized that disruption of the intrauterine environment by nutritional or chemical factors may influence the fetus, resulting in long-term adverse effects after birth and into adulthood^1^,^2^,^3^,^4^. By disrupting normal prenatal development, environmental factors and chemical exposures may lead to the fetal programming of adult disease, including cardiovascular disease^5^,^6^,^7^. It is also recognized that prenatal insults can have transgenerational effects^8^,^9^.

One of the most common chemicals that fetuses are exposed to is caffeine, which is a non-selective adenosine receptor antagonist^10^,^11^. Dietary caffeine comes from many sources including food, drinks and medication. Caffeine is present in foods such as chocolate, gum, beef jerky, and other foods including coffee-flavored yogurt^12^. Caffeinated beverages are widely consumed including tea, soft drinks, energy drinks, and coffee^13^. At serum concentrations achieved with human consumption, the physiological effects of caffeine are due to antagonism of adenosine action through competitive inhibition at the receptor level^10^,^11^. In addition, caffeine readily crosses the placenta to reach the fetus, where the half-life of caffeine is longer (12–24 hours) than in adults (2–4 hours) due to the absence of the enzyme CYP1A2 in placenta and fetus^11^,^14^,^15^.

Average caffeine intake is between 150 and 200 mg per day among women of child bearing age^14^. Caffeine consumption during the first month of pregnancy is reported by 60% of women, who often do not know that they are pregnant^14^. Caffeine consumption is associated with low birth weight and increased rates of spontaneous abortions^16^,^17^,^18^,^19^,^20^,^21^,^22^,^23^,^24^. Although, the notion that prenatal caffeine exposure is associated with low birth weight and other adverse effects is not accepted by all^14^,^25^, it is concerning enough that Nordic countries, the United Kingdoms, and the United States recommend that pregnant women limit their caffeine intake to less than 200 mg per day^26^,^27^.

Several reports indicate that *in utero* caffeine exposure can have long-term negative effects. In mice, maternal exposure to caffeine is associated with altered brain development and behavioral changes in neonatal and adult offspring^28^,^29^. In addition, *in utero* caffeine exposure is associated with high blood pressure, altered cardiac morphology, and reduced cardiac function in adult mice^30^,^31^,^32^. In humans, caffeine has long-lasting effects as well including reduced birth weight and growth in neonates and adverse behavior at 18 months^33^,^34^,^35^.

Increasing evidence shows that epigenetic processes can mediate heritable changes in gene expression without altering the DNA sequence and include DNA methylation, microRNA (miRNA), and histone modification^36^. Epigenetics also plays a critical role in mediating the fetal programming of adult disease^3^,^36^,^37^,^38^. Alterations in DNA methylation patterns are recognized to transduce *in utero* environmental stress into an increased risk for adult disease^39^. DNA methylation inhibits gene expression through suppressing transcription factor binding or recruiting histone deacetylases that cause chromatin condensation and gene inactivation^40^.

The DNA methylation state of the genome is changing throughout embryogenesis and two important DNA methylation events were targeted in our experiments. First, genomic DNA in the inner cell mass undergoes a rapid wave of demethylation after fertilization and this DNA methylation is re-established during embryogenesis from E3.5–9.5 in mice^41^,^42^,^43^. DNA methylation patterns can be altered by nutritional and environmental factors during this remethylation process resulting in long-lasting effects^43^,^44^,^45^,^46^. Second, primordial germ cells (PGCs) in mice go through specific DNA methylation remodeling events during embryogenesis^47^. PGCs migrate along the developing hindgut to the genital ridges^47^. During and after this migration (E10.5–13.5), PGC DNA actively undergoes a wave of demethylation^47^. This process is important for removing genomic imprints from the previous generation^47^,^48^. These epigenetic events can be affected by alteration within the intrauterine environment, including chemical exposures, nutritional deprivation, and oxygen availability. These effects are associated with adult diseases and transgenerational effects on health^8^,^9^,^49^,^50^,^51^,^52^,^53^.

We previously demonstrated that *in utero* caffeine exposure leads to long-term changes in the DNA methylation pattern in adult hearts^31^. In addition, we observed changes in expression of important DNA methylation enzymes, including DNA methyltransferases 1, 3a, 3b, in embryonic hearts exposed to caffeine *in utero*^54^. The timing of *in utero* caffeine exposure is important in this process, as it determines which DNA methylation process may be affected, and can determine which generations are affected, as well as the type of phenotypic changes induced in adulthood^7^.

Based on these data, we hypothesized that *in utero* caffeine exposure alters DNMT activity leading to altered DNA methylation patterns in adult tissue. We postulated that this altered DNA methylation pattern would lead to alter gene expression and cardiac function in adult hearts. In addition, we hypothesized that based on the timing of caffeine exposure and therefore which DNA methylation process is targeted, we would expect that different generations would be affected.

Our results confirmed our hypotheses as the results outlined in this report indicate *in utero* caffeine exposure leads to long-term altered effects on cardiac function, morphology, gene expression, and sensitivity to β-adrenergic stimulation in adult offspring. In addition, we demonstrate that *in utero* caffeine exposure leads to transgenerational effects on the heart in the F2 and F3 generations.

## Results

### The timing of in utero caffeine exposure influences long-term effects on cardiac function and morphology

At 12 weeks of age, F1 generation offspring of dams treated from E6.5–9.5 with caffeine did not have altered cardiac morphology or function (Table 1).

At 22 weeks after birth, F1 generation offspring of dams treated from E6.5–9.5 with caffeine had altered cardiac morphology, including increased interventricular septum (IVS) thickness during both systole and diastole, and increased left ventricle (LV) mass compared to vehicle controls (Table 1). In addition, we observed increased left ventricle internal diameter (LVID) and LV volume during both diastole and systole in the caffeine-treated group (Table 1).

At one year of age, F1 generation mice exposed to caffeine *in utero* from E6.5–9.5 showed altered cardiac morphology, as well as altered cardiac function that was characteristic of dilated cardiomyopathy. Hearts from the caffeine-treated group displayed systolic dysfunction including a 12.6% decrease in the left ventricular posterior wall thickness during systole (LVPW;s) and a 10.9% increase in LVID during systole (LVID;s), which led to a 24.9% increase in LV volume during systole (Table 1). These effects on cardiac morphology lead to reduced cardiac function, including a 17.3% decrease in % fractional shortening (%FS) and a 15.5% decrease in % ejection fraction (%EF; Table 1).

Indicating that the timing of caffeine exposure is critical for the observed effects on cardiac function and morphology, F1 adult offspring of dams exposed to caffeine from E10.5–13.5 did not display adverse effects on cardiac morphology or function in adulthood at 22 weeks or at 1 year of age (Table 2).

### In utero caffeine exposure leads to impaired β-adrenergic responsiveness in adulthood

At 12 weeks of age, caffeine-treated mice showed only a slight difference in morphology compared to vehicle controls after 20 minutes of dobutamine exposure, including an increase in IVS thickness during diastole (Table 3). At 12 weeks, dobutamine treatment had no effect on cardiac function in mice exposed to caffeine *in utero* (Table 3).

**Figure 1:**
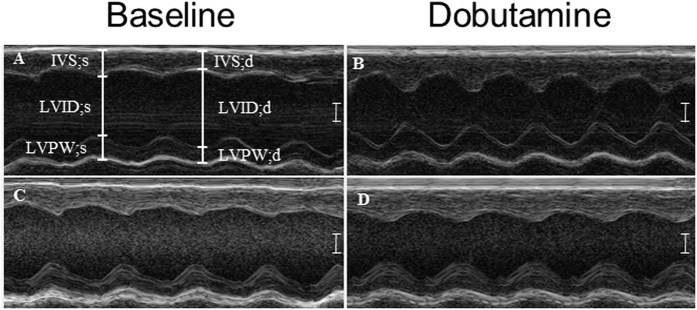
M-mode measurements before and after dobutamine treatment in 22 week old mice. For this analysis F1 generation mice from dams treated with vehicle or caffeine from E6.5–9.5 were used. Baseline echocardiograms were taken of (**A**) vehicle- and (**C**) caffeine-treated mice. 20 minutes after dobutamine another set of echocardiograms were taken of (**B**) vehicle- and (**D**) caffeine-treated mice. Data indicate that caffeine-exposed hearts have impaired response to β-adrenergic stimulation during systole and diastole. Measurements that were altered include interventricular septum (IVS;s); left ventricular posterior wall (LVPW;s, d); d, diastole; s, systole. N = 3–4, P ≤ 0.05. Scale bar = 1 mm.

At 22 weeks, hearts from the caffeine-treated group exhibited impaired responses to dobutamine treatment at the morphological and functional levels. We observed altered morphology in caffeine-treated hearts including decreased LVPW thickness (15.6%) during systole compared to controls (Fig. 1; Table 3). Changes in morphology were associated with reduced function including, decreased % EF (12.7%) and % FS (21.7%) compared to controls in response to dobutamine (Table 3). These data show that *in utero* caffeine exposure induces long-term adverse effects on the hearts ability to respond to β-adrenergic stimulation.

### In utero caffeine exposure leads to adverse transgenerational effects on adult cardiac morphology and function

In the F2 caffeine-treated group, we did not observe changes in cardiac morphology or function at 22 weeks of age, but there were significant adverse effects at 1 year of age (Table 2). At 1 year of age, the caffeine-treated group exhibited significant changes in cardiac morphology including decreased LVID, decreased LV volume and increased LVPW thickness during both systole and diastole (Table 2). These changes in morphology were associated with changes in cardiac function including a 10.6% increase in % FS (10.4%) and an 8.6% increase in % EF (Table 2). These changes in the caffeine-treated group are similar to those seen in hypertrophic cardiomyopathy with hyperdynamic function in humans^55^,^56^.

We also examined the F3 generation offspring, which were never directly exposed to caffeine. At 22 weeks, the F3 caffeine group displayed effects on morphology including increased LVPW thickness during systole and diastole compared to controls and a 7.7% increase in LV mass (Table 2). At 1 year of age, we observed that hearts of the F3 generation caffeine group had a 13.6% increase in LV mass compared to controls that did not coincide with an increase in body weight (Table 2).

### In utero caffeine exposure leads to altered cardiac gene expression in adulthood

**Figure 2:**
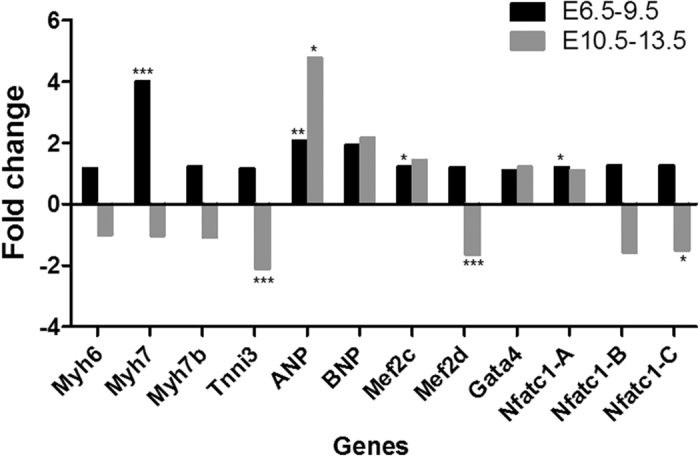
*In utero* caffeine exposure leads to altered cardiac gene expression in the F1 generation at 1 year of age. Early in utero exposure to caffeine from E6.5–9.5 caused increased RNA expression of *Myh7*, *ANP*, *Mef2c*, and *Nfatc-1A* in the left ventricle. Late in utero exposure from E10.5–13.5 caused altered expression of *Tnni3*, *ANP*, *Mef2d*, and *Nfatc1-C*. Fold change in gene expression between vehicle and caffeine treated mice was calculated with the 2^−∆∆CT^ method using *Gapdh* as the control gene. Significance was determined by Student’s t-test and a P ≤ 0.05 was determined to be significant. N = 4, *P ≤ 0.05, **P ≤ 0.01, and ***P ≤ 0.001.

We first assessed cardiac gene expression in F1 adult mice exposed to caffeine *in utero* from E6.5–9.5. At 1 year of age, we observed significant changes in several cardiac genes including a 4-fold increase in the structural gene *Myh7*, increases in transcription factors *Mef2c* and *Nfatc1-a*, and a 2-fold increase in the hormone *ANP* (Fig. 2). In F1 hearts exposed to caffeine from E10.5–13.5, we also observed changes in gene expression including *Tnni3*, *ANP*, *Mef2d*, and *Nfatc1-c* (Fig. 2). Other than ANP, the different exposure periods of caffeine caused long-term changes in expression of a different set of genes in F1 adult hearts (Fig. 2).

**Figure 3:**
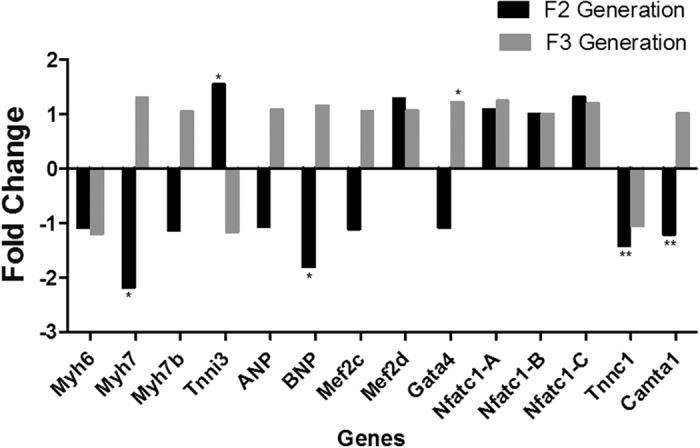
*In utero* caffeine exposure leads to altered cardiac gene expression in F2 and F3 generation at 1 year of age. qPCR analysis revealed that *in utero* caffeine treatment during E10.5–13.5 altered gene expression in the left ventricles of F2 generation offspring, including *Myh7*, *Tnni3*, *BNP*, *Tnnc1*, and *Camta1. In utero* caffeine treatment during E10.5–13.5 increased *Gata4* expression in the left ventricles of F3 generation offspring. Fold-change in gene expression between vehicle and caffeine treated mice was calculated with the 2^−∆∆CT^ method using *Gapdh* as the control gene. Significance was determined by Student’s t-test and a P ≤ 0.05 was determined to be significant. N = 5, *P ≤ 0.05, **P ≤ 0.01.

We examined gene expression in the F2 and F3 generation of offspring to determine if *in utero* caffeine exposure induces transgenerational changes in cardiac gene expression. In the F2 generation at 1 year, we observed significant changes in the structural cardiac genes *Myh7*, *Tnni3*, and *Tnnc1*, the transcription factor *Camta1*, and the hormone *BNP* (Fig. 3). In the F2 caffeine-treated hearts at 1 year, *Myh7* was down-regulated 2.2-fold (Fig. 3). In the F3 caffeine group, the only significant change in gene expression observed at 1 year of age was with *Gata4* (Fig. 3).

Next, we performed transcriptomic mRNA-sequencing on left ventricles for a more comprehensive analysis of gene expression in the F1 generation adult hearts at 1 year of age. The mapping efficiency of sequencing reads was about 80%, and the samples were well clustered by treatments (Supplementary Figure 1). Differential gene expression (DE) analysis by EdgeR software revealed that 85 genes were up-regulated, and 31 genes were down-regulated significantly (Supplementary Table 1).

Diseases and Bio Functions analysis by Ingenuity Pathway Analysis (IPA) revealed that pathways related to cell death and survival, cell morphology, cellular function and maintenance, and cell signaling were affected by *in utero* caffeine exposure from E6.5–9.5 (Table 4). In addition, many cardiovascular disease pathways were significantly enriched (Table 5). In particular, the myocardium infarction and permeability of vascular system pathways were inhibited (Table 5), possibly serving as a compensation mechanisms in response to the adverse effects caused by *in utero* caffeine treatment.

Further functional ontology analysis of genes altered by *in utero* caffeine exposure was conducted using the Database for Annotation, Visualization and Integrated Discovery (DAVID). DAVID analysis revealed that pathways related to immune response, antigen processing and presentation, GTP binding, and GTPase activity were affected by prenatal caffeine treatment (Supplementary Table 2). Also, mRNA expression of many cell surface molecules and signaling peptides were altered in caffeine-treated hearts (Supplementary Table 2). DAVID analysis of differentially expressed genes also identified sarcomere genes, including KAT2B (−1.68 fold), ACTA1 (2.69 fold), ABRA (1.76 fold), and MYOT (1.55 fold); genes involved in blood vessel development and blood circulation, including NPPB (4.28 fold), WARS (1.58 fold), GPX1 (1.55 fold), TNFRSF12A (1.93 fold), APOE (1.58 fold), and FGFBP3 (−117.24 fold); and genes related to membrane channel activity, including CACNG6 (−37.69 fold; calcium channel), KCNJ14 (2.39 fold; potassium channel), GRIN2C (3.06 fold; ionotropic glutamate receptor NMDA2C), and AQP4 (−15.92 fold; water channel).

## Discussion

**Figure 4:**
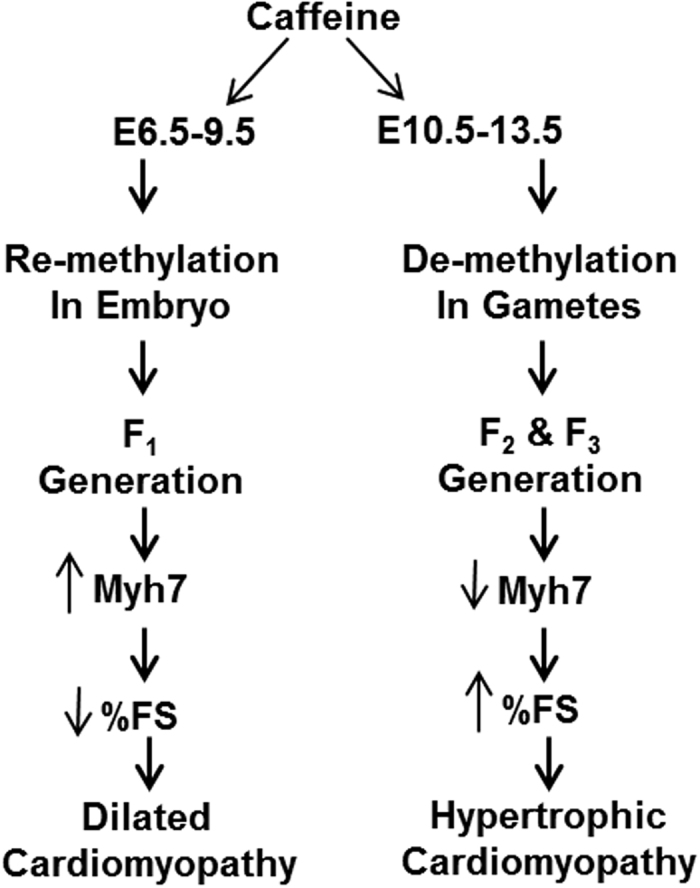
Timing of in utero caffeine exposure leads to different cardiac phenotypes in adult offspring. The timing of *in utero* caffeine treatment leads to differences in adult cardiac function, gene expression, and phenotype. Exposure to caffeine from E6.5–9.5 leads the F1 generation to develop dilated cardiomyopathy with decreased % FS and increased *Myh7* expression. *In utero* caffeine exposure from E10.5–13.5 leads to a hypertrophic cardiomyopathy in the F2 generation along with increased % FS and decreased *Myh7* expression.

We find that the timing of *in utero* caffeine exposure determines which generation is effected and the cardiac phenotype induced. Early embryonic exposure to caffeine leads to a dilated cardiomyopathy phenotype in the 1^st^ generation of offspring, and caffeine exposure later in development induces hypertrophic cardiomyopathy in the 2^nd^ generation and altered morphology in the 3^rd^ generation (Fig. 4). Collectively, these data show that caffeine exposure during fetal development has lasting effects on the heart, including reduced cardiac function and altered gene expression.

Although caffeine exposure is not a teratogen considered harmful to the developing fetus by many^14^,^25^, a recent Norwegian Mother and Child Cohort Study (MoBa) involving 59,123 pregnant women in Norway strongly supports the notion that caffeine consumption is associated with decreased birth weight, even at levels below recommended limits^35^. The decrease in birth weight associated with fetal caffeine exposure is small^35^, but studies demonstrate that reduced fetal growth is associated with higher instances of adult disease later in life, including cardiovascular disease^57^,^58^. Data from this report and our previous results^31^,^32^ support the notion that *in utero* caffeine exposure induces long-term effects on cardiac gene expression and function into adulthood.

In these studies, we examined CD-1 mice because they have more severe phenotypes compared to inbred strains in transgenerational studies^9^. We exposed CD-1 mice to caffeine early in gestation (E6.5–9.5), as before^31^,^32^, and observed a phenotype similar to that found in the inbred C57Bl6 strain^32^. The changes in cardiac morphology and function induced by *in utero* caffeine exposure in the F1 generation CD-1 mice are characteristics seen in dilated cardiomyopathy in humans including dilated ventricular cavity and systolic dysfunction^59^. The effects of caffeine on cardiac function in CD-1 mice were not observed until 1 year of age compared to the inbred C57Bl/6 strain that exhibited reduced cardiac function at 10 weeks of age^32^. Thus, the CD-1 strain develops cardiac dysfunction later in adulthood compared to the inbred strain, but the effects are more severe^31^,^32^.

Based on our previous data^31^,^32^, we hypothesized that *in utero* caffeine exposure disrupts the DNA re-methylation event that occurs from E3.5–9.5 throughout the embryo^43^. Our results support this hypothesis as exposure to caffeine later in development from E10.5–13.5 had no effect on cardiac function or morphology in the F1 generation at either 22 weeks or 1 year of age. These results indicate that the timing of caffeine exposure is critical to inducing long-term effects in adult hearts of the F1 generation.

During the E10.5–13.5 developmental window, primordial germ cells are migrating, maturing, and going through a process of demethylation^43^. Thus, we hypothesized that caffeine could interfere with this demethylation process, which could lead to altered cardiac function in the F2 (germ cells at the time of exposure) and possibly the F3 (not directly exposed to caffeine) generations.

The effects of *in utero* caffeine exposure on adult cardiac function and morphology in the F2 generation offspring were opposite to those seen in the F1 generation exposed to caffeine from E6.5–9.5. The F2 caffeine hearts at 1 year displayed decreased LVID;s, increased LVPW;s thickness, and increased FS, which are characteristic of hypertrophic cardiomyopathy, including asymmetric left ventricular thickening, increased LV cavity, and hyperdynamic function^55^,^56^.

The F3 generation caffeine hearts had altered morphology and increased mass. Although caffeine induces effects on the heart in the F3 generation, they are less severe and they do not lead to altered cardiac function.

In addition, we observed that *in utero* treated (from E6.5–9.5) caffeine hearts had impaired responses to dobutamine stimulation, indicating that caffeine treated hearts may have a reduced ability to respond to or sense β-adrenergic stimulation. This impaired response could have a significant clinical impact as β-adrenergic agonists are given to patients with failing hearts to increase cardiac output.

We find that *in utero* caffeine exposure has long-lasting effects on cardiac gene expression in adult offspring of the F1, F2, and F3 generation. The most significant change in gene expression observed was with the *Myh7* gene. MYH7 is generally an embryonic and prenatal form of cardiac myosin that is greatly reduced in adult murine hearts, but *Myh7* expression is activated in adult hearts during cardiac stress and heart failure in mice^60^,^61^. In F1 generation mice exposed to caffeine from E6.5–9.5, we observed an increase in *Myh7* expression. The increase in *Myh7* expression is indicative of the phenotype induced by caffeine as increased *Myh7* expression is associated with cardiomyopathy and heart failure^61^,^62^. Two other genes, *Tnni3* and *Mef2c,* were also altered in adult F1 hearts when exposed to caffeine early in embryogenesis. These genes are associated with dilated cardiomyopathy^63^,^64^,^65^ and we have demonstrated previously that their expression is sensitive to caffeine exposure^54^. In addition, we observe increased ANP expression in adult F1 hearts which is also increased in human idiopathic dilated cardiomyopathy^66^. From our RNAseq data we identify new genes that we had not previously associated with caffeine exposure and cardiomyopathy, including *Nppb* which is up regulated in F1 hearts at one year and in dilated cardiomyopathy^67^, and *Mylk4* which is down regulated in our caffeine exposed F1 hearts and in human dilated cardiomyopathy patient left ventricle tissue^68^.

In contrast, we observed that F2 generation mice exposed to *in utero* caffeine from E10.5–13.5 had reduced expression of *Myh7.* This opposite effect on *Myh7* gene expression compared to the F1 generation mice exposed to caffeine from E6.5–9.5 correlates with the opposite effect that caffeine has on adult heart function in the F2 generation. These data indicate that *Myh7* expression is sensitive to caffeine exposure and may be predictive of cardiac dysfunction in adult hearts (Fig. 4).

In conclusion, we find that *in utero* caffeine exposure has long-term effects on cardiac morphology, function, and gene expression. We find that long-term effects of caffeine exposure *in utero* are determined in part by the timing of exposure (Fig. 4). We show that caffeine may interfere with the hearts ability to respond to stress, as caffeine treated mice had an impaired response to β-adrenergic stimulation. In addition, we demonstrate that caffeine has transgenerational effects on the F2 generation including altered cardiac morphology, function, and gene expression. Transgenerational effects of caffeine are also observed in the F3 generation. As such, these observations are of potential major clinical significance to the thousands of women who consume caffeine during pregnancy.

## Methods

### Animals

All animal experiments were approved by the Institutional Animal Care and Use Committee (IACUC) at the University of Florida and all methods were carried out in accordance with approved guidelines. Pregnant CD-1 dams (F0 generation) were treated with 0.9% NaCl (vehicle) or 20 mg/kg caffeine in vehicle via i.p. injection. CD-1 mice were used as they have been demonstrated to have more severe transgenerational effects on health in adulthood than inbred strains when exposed to endocrine disruptors *in utero*^9^. This dose was chosen because we previously demonstrated that it leads to long-term effects on cardiac function and results in a serum level equivalent to 2–4 cups of coffee in humans^31^,^32^. Dams were treated once daily for four days either from E6.5–9.5 or E10.5–13.5. We chose these exposure times because they coincide with two important DNA methylation transition events during embryogenesis, including a large scale DNA remethylation of the genome that occurs from E3.5–9.5, and genome-wide demethylation that occurs during PGC development from E10.5–13.5 43.

Our previous analysis indicated that there was no difference in the effects of *in utero* caffeine exposure on cardiac function between males and females^32^. In addition, we observed elevated % body fat levels in males exposed to caffeine *in utero*^32^, therefore we only examined male offspring in this study.

The F1 generation (embryos exposed *in utero*), F2 generation (exposed gametes within the F1 generation embryos) and F3 generation (naïve to caffeine exposure) were examined up to one year of age (Tables 6 and 7). We generated the F2 generation by mating F1 generation mice at 10–12 weeks of age that had been similarly treated with either caffeine or vehicle, taking care not to mate siblings or cousins. Next, we generated the F3 generation of mice, which were naïve to caffeine exposure, by mating F2 generation mice that were similarly treated.

### Statistical Analysis

Data are presented as mean ± the standard error of the mean (SEM). Analysis was performed with the statistics software package included with Microsoft Excel (Microsoft, Redmond, WA, USA) and GraphPad Prism 6.0 (GraphPad Software Inc., La Jolla, CA, USA).

### Echocardiography

Cardiac function of male offspring was assessed using echocardiography, as described^31^,^32^,^69^. Briefly, offspring were anesthetized with a continuous flow of isoflurane administered via nosecone and anesthesia levels were regulated to maintain heart rates between 400 and 500 beats per minute. Transthoracic 2D M-mode echocardiography was performed using a 30-MHz probe with the Vevo 770 (Visualsonics, Toronto, ON, Canada) small animal ultrasound device^32^.

Ultrasound analysis of cardiac function was performed after administration of dobutamine, a β-adrenergic agonist, in order to stress the heart. Dobutamine, an inotropic agent, induces a more forceful contraction of heart muscle, which increases the LVPW thickness during systole leading to increased % FS and % EF^70^. After baseline echocardiograms were obtained, mice were injected with 1.5 mg/kg dobutamine via i.p. Echocardiograms were retaken 20 minutes after dobutamine injection. Echocardiography and analysis of results were performed blinded. Analyses of the effects of dobutamine were determined by comparing echocardiograms at baseline and 20 minutes. Based on a pilot experiment that examined cardiac function 5, 10, 15, and 20 minutes after dobutamine injection, we determined that the results at 20 minutes were the most significant compared to baseline.

For echocardiography, 1–5 offspring from 3–6 dams were analyzed. Statistical comparisons between groups for echocardiography were performed with Student’s t-test assuming equal variance. For Tables 1, 2, and 3 N = number of dams treated. For Tables 1, 2, and 3 measurements for offspring from one dam were averaged and the averages for each litter were used to calculate significance. For Table 3, N = the number of mice treated with dobutamine. P ≤ 0.05 was considered to be statistically significant.

### Quantitative real-time PCR (qPCR) analysis

For qPCR, 1–2 offspring were examined from 4–5 different dams. Results from offspring of the same dam were averaged before determining significance and fold change. Total RNA from left ventricles of adult male offspring was extracted with RNeasy Plus Mini Kit (Qiagen), according to the manufacturer’s protocol. cDNA was synthesized using iScript cDNA Synthesis Kit (Bio-Rad, Hercules, CA, USA). For most cardiac genes, published primer pairs were used^71^. *Camta1* forward: TCCTTATCCAGAGCAAATTCCGA; *Camta1* reverse: CACACTTCTTGTAACTCCGGTAG. *Myh6* (PPM04500A) and *Myh7* (PPM67019A) primers were proprietary sequences designed by Qiagen SABiosciences. *Gapdh* primers were used as an internal control. SYBR^®^Green PCR Master Mix (Life Technologies Applied Biosystems^®^) was used to perform qPCR analysis in a GeneAmp 7300 Real Time PCR System^71^. Each sample was measured in two separate reactions on the same plate. Amplification efficiencies of the target genes and *Gapdh* primer pairs were tested to ensure that they were not statistically different. Differences in expression between the treatment groups were calculated with the 2^−∆∆CT^ method^54^. Statistical significance was determined by Student’s t-test analysis on the ΔCT values, thus error bars are not included on the gene expression graphs (Figs 2 and 3).

### Illumina transcriptomic RNA sequencing (RNA-Seq)

Total RNA was isolated from the left ventricles of male 1 year-old mice treated with 20 mg/kg caffeine at E6.5–9.5 by using the RNeasy Plus Mini kit (Qiagen). mRNA was isolated from total RNA using NEXTflex™ Poly(A) Beads (Bioo Scientific, Austin, TX, USA). Sequencing libraries were prepared with the NEBNext^®^ mRNA Library Prep Master Mix Set for Illumina (NEB, Ipswich, MA, USA) and the NEBNext Multiplex Oligos for Illumina (NEB). Illumina-adapted libraries (n = 3/treatment) were pooled at equal molar ratio and sequenced with one High Output 1 × 75 cycles run on a NextSeq500 sequencer (Illumina, San Diego, CA, USA). All RNA-Seq data were uploaded to the Gene Expression Omnibus (GEO), and the accession number is GSE79013.

### RNA-Seq data analysis for differential gene expression

The fastq files generated from RNA-Seq were uploaded to the UF Research Computing Galaxy instance developed by the University of Florida^72^. The data were cleaned with the FastQC program and mapped to the mouse genome (mm10) with the Tophat2 tool. Counting of RNA-seq reads were performed with HTSeq^73^. Differential expression (DE) of genes between treatments was analyzed using R package EdgeR^74^, with Ensembl Mus_GRCm38.79.gtf as the reference annotation. Genes with false discovery rate (FDR) less than 0.05 and absolute fold change greater than 1.5 were considered as significant. EdgeR was also used to calculate the principle component (x-axis) analysis to determine the variance between the vehicle- and caffeine-treated groups (Supplementary Figure 1). Functional ontology was conducted using the Database for Annotation, Visualization and Integrated Discovery (DAVID) and IPA (Qiagen), and the significance was determined as described for each software package^75^,^76^. The significance criterion for pathway analysis is p ≤ 0.05 with Benjamini Hochberg correction.

## Additional Information

**How to cite this article**: Fang, X. *et al.* In Utero Caffeine Exposure Induces Transgenerational Effects on the Adult Heart. *Sci. Rep.* **6**, 34106; doi: 10.1038/srep34106 (2016).

## Supplementary Material

Supplementary Information

## Figures and Tables

**Table 1:** Echocardiography analysis of adult F1 generation mice exposed to caffeine or vehicle as embryos *in utero* from E6.5–9.5.

Treatment	Age	IVS;d	LVID;d	LVPW;d	LV Vol;d	IVS;s	LVID;s	LVPW;s	LV Vol;s	% EF	% FS	LV Mass	Heart Rate	Weight	N
E6.5–9.5		mm	mm	mm	ul	mm	mm	mm	ul	%	%	mg	BPM	g	
Vehicle	**12 Weeks**	0.74	4.77	0.84	106.50	1.01	3.56	1.17	53.69	49.90	25.53	124.70	481.57	39.85	4
Caffeine	**12 Weeks**	0.80	4.60	0.89	97.92	1.10	3.29	1.23	45.23	54.76	28.69	128.07	475.44	42.98	4
P-value		0.07	0.08	0.18	0.08	0.10	0.06	0.24	0.09	0.09	0.08	0.36	0.42	0.08	
E6.5–9.5															
Vehicle	**22 Weeks**	0.84	4.63	0.93	98.97	1.15	3.25	1.30	43.26	56.58	29.81	137.03	503.31	53.54	3
Caffeine	**22 Weeks**	0.92*	4.81*	0.92	108.31*	1.25	3.50*	1.26	51.65*	52.72	27.35	154.51*	499.41	53.86	4
P-value		0.02	0.01	0.46	0.01	0.06	0.04	0.28	0.05	0.14	0.15	0.04	0.45	0.46	
E6.5–9.5															
Vehicle	**1 year**	0.95	4.68	1.07	102.53	1.33	3.30	1.49	44.87	56.47	29.66	169.77	495.54	62.13	4
Caffeine	**1 year**	0.96	4.94	1.06	115.91	1.30	3.70*	1.32*	59.77*	48.89*	25.28*	183.34	469.46	59.36	4
P-value		0.45	0.11	0.42	0.12	0.35	0.04	0.02	0.04	0.02	0.02	0.26	0.18	0.34	

IVS, interventricular septum; LVID, left ventricle internal diameter; LVPW, left ventricular posterior wall; LV, left ventricle; FS, fraction shortening; Vol, volume; EF, ejection fraction; d, diastole; s, systole. Units: IVS, LVID, LVPW are measured in millimeters (mm); LV vol is measured in milliliters (ml); LV mass is measured in milligrams (mg). ^*^P-value ≤ 0.05. P-values were calculated by Student’s t-test.

**Table 2:** Echocardiography analysis of F1, F2, and F3 generation adult offspring of F0 dams exposed to vehicle or caffeine.

	Age/Generation	IVS;d	LVID;d	LVPW;d	LV Vol;d	IVS;s	LVID;s	LVPW;s	LV Vol;s	% EF	% FS	LV Mass	Heart Rate	Weight	N
E10.5-13.5	**22 Weeks**	mm	mm	mm	ul	mm	mm	mm	ul	%	%	mg	BPM	g	
Vehicle	**F1**	1.00	4.47	0.94	92.01	1.37	3.24	1.24	43.55	53.71	28.02	147.37	521.75	50.83	5
Caffeine	**F1**	0.94	4.61	0.98	99.24	1.26	3.49	1.24	52.95	47.11	24.11	150.62	493.81	50.97	4
P-value		0.21	0.25	0.11	0.21	0.14	0.20	0.50	0.16	0.15	0.16	0.42	0.16	0.49	
E10.5-13.5	**1 year**														
Vehicle	**F1**	1.03	4.58	0.98	96.47	1.34	3.26	1.34	43.46	55.47	28.94	157.57	496.03	52.51	5
Caffeine	**F1**	0.90	4.84	1.01	110.90	1.25	3.49	1.38	52.46	54.03	28.19	164.50	473.44	61.33	4
P-value		0.08	0.14	0.27	0.13	0.16	0.21	0.29	0.20	0.37	0.39	0.32	0.15	0.08	
E10.5–13.5	**22 Weeks**														
Vehicle	**F2**	0.88	4.62	0.94	98.84	1.21	3.36	1.25	46.72	53.01	27.42	142.55	494.17	52.16	5
Caffeine	**F2**	0.84	4.59	0.95	97.20	1.16	3.29	1.29	44.35	54.29	28.36	137.13	471.52	50.06	4
P-value		0.23	0.42	0.48	0.41	0.23	0.31	0.33	0.32	0.37	0.36	0.28	0.20	0.30	
E10.5–13.5	**1 year**														
Vehicle	**F2**	0.92	5.00	1.00	118.72	1.29	3.66	1.34	57.24	52.09	26.89	173.76	523.84	60.10	5
Caffeine	**F2**	0.89	4.75*	1.11*	105.32*	1.25	3.32*	1.50*	45.54*	57.01*	30.07*	169.31	526.52	59.62	7
P-value		0.22	0.03	0.01	0.03	0.24	0.02	0.01	0.02	0.04	0.04	0.33	0.44	0.47	
E10.5–13.5	**22 Weeks**														
Vehicle	**F3**	0.92	4.60	0.94	97.37	1.28	3.32	1.25	45.24	53.67	27.75	145.25	518.58	51.71	5
Caffeine	**F3**	0.92	4.77	0.96*	106.35	1.28	3.40	1.33*	48.24	54.95	28.66	157.30*	498.39	51.35	4
P-value		0.31	0.09	0.04	0.09	0.37	0.33	0.01	0.31	0.23	0.21	0.05	0.39	0.31	
E10.5–13.5	**1 year**														
Vehicle	**F3**	1.00	4.70	1.06	102.75	1.42	3.28	1.48	44.50	57.26	30.39	174.27	554.53	62.73	5
Caffeine	**F3**	0.95	5.07	1.15	125.27	1.42	3.28	1.48	67.29	49.37	25.72	201.70*	552.16	63.04	4
P-value		0.26	0.18	0.08	0.15	0.27	0.20	0.16	0.18	0.26	0.28	0.04	0.44	0.35	

Only the F1 generation embryos were exposed to vehicle or caffeine *in utero* from E10.5-13.5. For abbreviations please see Table 1. ^*^P-value ≤ 0.05. P-values were calculated by Student’s t-test.

**Table 3:** Echocardiography analysis 20 minutes after dobutamine treatment of CD-1 mice exposed to caffeine or vehicle *in utero* from E6.5–9.5.

Treatment	**Age**	IVS;d	LVID;d	LVPW;d	LV Vol;d	IVS;s	LVID;s	LVPW;s	LV Vol;s	% EF	% FS	LV Mass	Heart Rate	N
E6.5–9.5		mm	mm	mm	ul	mm	mm	mm	ul	%	%	mg	BPM	
Vehicle	**12 Weeks**	0.82	4.22	0.99	80.06	1.35	2.35	1.63	20.62	75.31	44.59	122.21	489.36	4
Caffeine	**12 Weeks**	0.89*	4.03	1.02	71.98	1.42	2.21	1.58	17.54	76.68	45.67	121.04	503.48	4
P-value		0.01	0.09	0.31	0.08	0.07	0.15	0.33	0.14	0.29	0.31	0.43	0.17	
E6.5–9.5														
Vehicle	**22 Weeks**	0.92	4.25	1.09	81.43	1.59	2.11	1.78	15.38	81.65	50.53	142.17	485.50	3
Caffeine	**22 Weeks**	0.95	4.15	0.99*	77.45	1.45*	2.44	1.54*	22.24	72.42*	41.53*	130.98	529.10*	3
P-value		0.15	0.33	0.01	0.35	0.01	0.12	0.001	0.15	0.03	0.02	0.20	0.01	

For abbreviations please see Table 1. ^*^P-value ≤ 0.05. P-values were calculated by Student’s t-test.

**Table 4:** Altered cellular morphology and function pathways in adult F1 mice treated with caffeine during E6.5–10.5.

	p-value	Activation z-score^a^	# of molecules
Cell death and survival			
Cytolysis	6.79E-04	−1.034	7
Proliferation of cells	2.11E-03	−0.513	37
Cell death	2.40E-03	−0.323	34
Cell morphology, cellular function and maintenance			
Nucleation of filaments	9.91E-04	—	3
Cellular homeostasis	3.38E-03	0.297	19
Morphology of cells	5.15E-03	—	22
Generation of filaments	6.22E-03	—	2
Cell signaling			
Activation of cells	2.76E-05	−0.500	18
Quantity of Ca^2+^	8.31E-03	−0.240	7

^a^A positive z-score means the pathway is activated, and a negative z-score indicates the pathway is inhibited.

**Table 5:** Altered cardiovascular disease pathways in adult F1 mice treated with caffeine during E6.5–9.5.

Pathways	p-value	Activation z-score^a^	Genes
Infarction	3.47E-06	−2.750	ACTA1,APOE,C1QA,CD55,FSTL1,GDF15,GPX1,HLA-A,PTGDS,STAT1,TNFRSF12A
Myocardial infarction	8.08E-04	−1.992	ACTA1,APOE,CD55,FSTL1,GDF15,STAT1
Permeability of vascular system	8.44E-03	−1.969	APOE,AQP4,FGFBP3,TNFRSF12A
Size of infarct	3.31E-06	−1.673	APOE,C1QA,GDF15,GPX1,HLA-A,PTGDS,STAT1,TNFRSF12A
Vasculogenesis	6.05E-03	−1.109	ABRA,APOE,C1QA,GPX1,HLA-DQB1,KLK3,SEMA3B,STAT1,THBS4,TNFRSF12A,WARS
Adhesion of endothelial cells	6.33E-03	−0.293	C1QA,CD274,GDF15,STAT1
Lesioning of aorta	9.10E-07		APOE,GPX1,NPC1
Peripheral vascular disease	8.48E-03		APOE,AQP4,CFH,GPX1,MARK1,SEMA3B,TNFRSF12A
Size of vascular lesion	7.61E-03		APOE,C1QA,GPX1
Advanced stage peripheral arterial disease	6.89E-03		APOE,CFH,MARK1,SEMA3B
Thickness of heart ventricle	6.70E-03		APOE,KLK3
Cellularity of atherosclerotic lesion	2.05E-05		APOE,NPC1
Function of cardiovascular system	1.18E-03		ABRA,APOE,AQP4,CD55,CFP,GDF15,GPX1,KLK3
Thrombosis	1.16E-02		APOE,C1QA,CFH,GDF15
Mass of left ventricle	1.05E-02		KLK3,THBS4

^a^A positive z-score means the pathway is activated, and a negative z-score indicates the pathway is inhibited.

**Table 6:** Timing of analysis performed on F1 offspring exposed to vehicle or caffeine *in utero* from E6.5–9.5.

Dam	F1 Offspring		
	Experimental Analysis		
Treatment/Timing	12 Weeks	22 Weeks	52 Weeks
Vehicle/E6.5–9.5 Caffeine/E6.5–9.5	1) Echocardiography	1) Echocardiography	1) Echocardiography
	2) Dobutamine Treatment	2) Dobutamine Treatment	2) Gene Expression
			4) RNA Seq.
			5) Histology

**Table 7:** Timing of analysis performed on F1, F2, and F3 offspring exposed to vehicle or caffeine in utero from E10.5–13.5.

Dam	F1 Offspring		F2 Offspring		F3 Offspring	
Treatment/Timing	Analysis		Analysis		Analysis	
	22 Weeks	52 Weeks	22 Weeks	52 Weeks	22 Weeks	52 Weeks
Vehicle/E10.5–13.5 Caffeine/E10.5–13.5	1) Echos	1) Echos 2) Gene Expression 3) Histology	1) Echos	1) Echos 2) Gene Expression 3) Histology	1) Echos	1) Echos 2) Gene Expression 3) Histology
