# Supplementary material for: In Utero Caffeine Exposure Induces Transgenerational Effects on the Adult Heart
Source: Sci Rep. 2016 Sep 28;6:34106. doi: 10.1038/srep34106 (PMC5039698; doi:10.1038/srep34106)
Supplement: Supplementary Information [file srep34106-s1.doc]

**Supplementary Information for “In Utero Caffeine Exposure Induces Transgenerational Effects on the Adult Heart.” Xiefan Fang, Ryan R. Poulsen, Scott A. Rivkees, and Christopher C. Wendler**

**
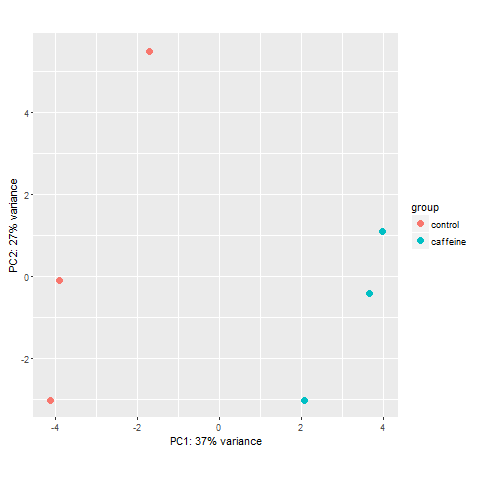
**

**Supplementary Figure 1.** Principal component analysis (PCA) was performed on the RNA-Seq data. TheRNA-seq samples from the F1 generation of adult left ventricles were well-separated based on treatment by the first principal component (X-axis) analysis.

**Supplementary Table 1.** Differentially expressed genes in left ventricles of F1 generation 1 year old mice treated *in utero* from E6.5-9.5.

| **Gene name** | **Description** | **Fold** | **FDR** |
| --- | --- | --- | --- |
| Gm4841 | predicted gene 4841 | 27.12 | 4.27E-13 |
| Gm22513 | predicted gene, 22513 | 25.14 | 0.010816 |
| Tspan8 | tetraspanin 8 | 13.98 | 0.013006 |
| BC023105 | cDNA sequence BC023105 | 11.24 | 0.000405 |
| Gm8810 | predicted gene 8810 | 10.18 | 0.003223 |
| Tgtp2 | T cell specific GTPase 2 | 8.36 | 4.30E-08 |
| H2-Eb1 | histocompatibility 2, class II antigen E beta | 8.11 | 2.43E-05 |
| Dupd1 | dual specificity phosphatase and pro isomerase domain containing 1 | 7.58 | 0.014527 |
| Snord17 | small nucleolar RNA, C/D box 17 | 6.70 | 4.83E-05 |
| H2-K2 | histocompatibility 2, K region locus 2 | 6.43 | 0.006966 |
| Cxcl13 | chemokine (C-X-C motif) ligand 13 | 6.43 | 0.013532 |
| Gm25360 | predicted gene, 25360 | 5.79 | 0.039398 |
| Igsf23 | immunoglobulin superfamily, member 23 | 5.77 | 0.00144 |
| Igtp | interferon gamma induced GTPase | 5.04 | 3.87E-07 |
| Igkc | immunoglobulin kappa constant | 4.87 | 0.021282 |
| Rpph1 | ribonuclease P RNA component H1 | 4.60 | 1.52E-09 |
| Tceal7 | transcription elongation factor A (SII)-like 7 | 4.59 | 0.020831 |
| Gm12250 | predicted gene 12250 | 4.45 | 0.004399 |
| Rmrp | RNA component of mitochondrial RNAase P | 4.29 | 1.38E-06 |
| Nppb | natriuretic peptide type B | 4.28 | 1.61E-20 |
| Iigp1 | interferon inducible GTPase 1 | 4.09 | 1.84E-10 |
| Irgm2 | immunity-related GTPase family M member 2 | 3.87 | 2.26E-09 |
| Rprl3 | ribonuclease P RNA-like 3 | 3.85 | 0.000606 |
| Rn7sk | RNA, 7SK, nuclear | 3.81 | 1.44E-06 |
| Gm11967 | predicted gene 11967 | 3.66 | 0.011458 |
| Ifi47 | interferon gamma inducible protein 47 | 3.62 | 0.000412 |
| Gbp3 | guanylate binding protein 3 | 3.58 | 5.52E-08 |
| Vgll2 | vestigial like 2 homolog (Drosophila) | 3.21 | 1.08E-06 |
| Nlrc5 | NLR family, CARD domain containing 5 | 3.20 | 0.006196 |
| Grin2c | glutamate receptor, ionotropic, NMDA2C (epsilon 3) | 3.06 | 0.001608 |
| AA474408 | expressed sequence AA474408 | 2.85 | 0.001608 |
| Grk5 | G protein-coupled receptor kinase 5 | 2.83 | 0.000181 |
| Gm25911 | predicted gene, 25911 | 2.82 | 0.010505 |
| Gdf15 | growth differentiation factor 15 | 2.80 | 0.005753 |
| Psmb9 | proteasome (prosome, macropain) subunit, beta type 9 (large multifunctional peptidase 2) | 2.77 | 0.001473 |
| H2-Aa | histocompatibility 2, class II antigen A, alpha | 2.72 | 0.00048 |
| Folr2 | folate receptor 2 (fetal) | 2.70 | 0.000994 |
| Acta1 | actin, alpha 1, skeletal muscle | 2.69 | 1.09E-09 |
| Cfp | complement factor properdin | 2.67 | 0.022477 |
| Gbp4 | guanylate binding protein 4 | 2.61 | 8.31E-05 |
| Cd274 | CD274 antigen | 2.49 | 0.004295 |
| Klk1b26 | kallikrein 1-related petidase b26 | 2.47 | 0.026987 |
| Cd55 | CD55 molecule, decay accelerating factor for complement | 2.44 | 0.00283 |
| Gbp7 | guanylate binding protein 7 | 2.43 | 3.31E-06 |
| H2-Ab1 | histocompatibility 2, class II antigen A, beta 1 | 2.40 | 0.02953 |
| Kcnj14 | potassium inwardly-rectifying channel, subfamily J, member 14 | 2.39 | 0.007025 |
| Yam1 | predicted gene, 42418 | 2.36 | 0.000854 |
| Trappc6a | trafficking protein particle complex 6A | 2.31 | 0.024557 |
| Gbp2b | guanylate binding protein 2b | 2.31 | 0.004399 |
| Lyrm7 | LYR motif containing 7 | 2.21 | 0.027544 |
| Nrgn | neurogranin | 2.19 | 0.039398 |
| Gbp2 | guanylate binding protein 2 | 2.14 | 5.28E-05 |
| Gck | glucokinase | 2.14 | 0.027544 |
| Pdlim2 | PDZ and LIM domain 2 | 2.06 | 0.016233 |
| Cobl | cordon-bleu WH2 repeat | 2.04 | 0.010277 |
| Tppp3 | tubulin polymerization-promoting protein family member 3 | 2.00 | 0.013532 |
| Irf9 | interferon regulatory factor 9 | 1.97 | 0.013006 |
| Csrp1 | cysteine and glycine-rich protein 1 | 1.95 | 0.013338 |
| C1qa | complement component 1, q subcomponent, alpha polypeptide | 1.95 | 0.016233 |
| Mad2l1bp | MAD2L1 binding protein | 1.95 | 0.039398 |
| Tnfrsf12a | tumor necrosis factor receptor superfamily, member 12a | 1.93 | 1.45E-06 |
| Lyz2 | lysozyme 2 | 1.90 | 0.030211 |
| Nr1d1 | nuclear receptor subfamily 1, group D, member 1 | 1.89 | 0.033245 |
| Thbs4 | thrombospondin 4 | 1.89 | 0.003223 |
| Bambi | BMP and activin membrane-bound inhibitor | 1.87 | 0.015204 |
| Stat1 | signal transducer and activator of transcription 1 | 1.87 | 0.026987 |
| Gm23935 | predicted gene, 23935 | 1.81 | 2.24E-05 |
| Mir6236 | microRNA 6236 | 1.79 | 0.000457 |
| Igfbp6 | insulin-like growth factor binding protein 6 | 1.77 | 0.028847 |
| Ptgds | prostaglandin D2 synthase (brain) | 1.77 | 0.006966 |
| Abra | actin-binding Rho activating protein | 1.76 | 0.002418 |
| Cfh | complement component factor h | 1.76 | 0.001351 |
| Lars2 | leucyl-tRNA synthetase, mitochondrial | 1.67 | 0.010505 |
| Gm26917 | predicted gene, 26917 | 1.66 | 0.000291 |
| Crip1 | cysteine-rich protein 1 (intestinal) | 1.64 | 0.012386 |
| Gm15564 | predicted gene 15564 | 1.63 | 0.021282 |
| Apoe | apolipoprotein E | 1.58 | 0.034639 |
| Wars | tryptophanyl-tRNA synthetase | 1.58 | 0.014689 |
| Myot | myotilin | 1.55 | 0.039398 |
| Gpihbp1 | GPI-anchored HDL-binding protein 1 | 1.55 | 0.039668 |
| Gpx1 | glutathione peroxidase 1 | 1.54 | 0.019785 |
| Fstl1 | follistatin-like 1 | 1.52 | 0.025131 |
| Bri3 | brain protein I3 | 1.52 | 0.014119 |
| Cpxm2 | carboxypeptidase X 2 (M14 family) | 1.51 | 0.025131 |
| Plekho1 | pleckstrin homology domain containing, family O member 1 | 1.48 | 0.046859 |
| Entpd5 | ectonucleoside triphosphate diphosphohydrolase 5 | -1.48 | 0.039398 |
| Nckap1 | NCK-associated protein 1 | -1.56 | 0.037399 |
| Ppm1k | protein phosphatase 1K (PP2C domain containing) | -1.56 | 0.032578 |
| Lsm14b | LSM14 homolog B (SCD6, S. cerevisiae) | -1.58 | 0.02723 |
| St3gal5 | ST3 beta-galactoside alpha-2,3-sialyltransferase 5 | -1.62 | 0.034639 |
| Agtpbp1 | ATP/GTP binding protein 1 | -1.63 | 0.039398 |
| Kat2b | K(lysine) acetyltransferase 2B | -1.68 | 0.005217 |
| Rgma | repulsive guidance molecule family member A | -1.73 | 0.039398 |
| H2-D1 | histocompatibility 2, D region locus 1 | -1.76 | 0.039398 |
| Sspn | sarcospan | -1.82 | 0.00514 |
| Mboat2 | membrane bound O-acyltransferase domain containing 2 | -1.84 | 0.032578 |
| Sema3b | sema domain, immunoglobulin domain (Ig), short basic domain, secreted, (semaphorin) 3B | -1.90 | 0.032163 |
| Dnajc1 | DnaJ (Hsp40) homolog, subfamily C, member 1 | -1.96 | 0.018582 |
| Cyp2d22 | cytochrome P450, family 2, subfamily d, polypeptide 22 | -1.98 | 0.020429 |
| Man2a2 | mannosidase 2, alpha 2 | -2.02 | 0.033719 |
| Hlf | hepatic leukemia factor | -2.11 | 0.005147 |
| Tspan5 | tetraspanin 5 | -2.21 | 0.011892 |
| Fign | fidgetin | -2.22 | 0.037399 |
| Mark1 | MAP/microtubule affinity regulating kinase 1 | -2.25 | 0.030008 |
| Pde7b | phosphodiesterase 7B | -2.31 | 0.008159 |
| Gm10524 | predicted gene 10524 | -2.36 | 0.040637 |
| Mylk4 | myosin light chain kinase family, member 4 | -2.38 | 0.013006 |
| Npc1 | Niemann-Pick type C1 | -2.69 | 0.002107 |
| Irx2 | Iroquois related homeobox 2 (Drosophila) | -3.31 | 0.001203 |
| Gm10435 | predicted gene 10435 | -3.57 | 0.035394 |
| Ankrd63 | ankyrin repeat domain 63 | -10.47 | 0.00283 |
| Aqp4 | aquaporin 4 | -15.92 | 0.003731 |
| Cntn2 | contactin 2 | -19.01 | 0.001116 |
| Cacng6 | calcium channel, voltage-dependent, gamma subunit 6 | -37.69 | 0.015204 |
| Fgfbp3 | fibroblast growth factor binding protein 3 | 117.24 | 0.021064 |
| Hmgb1-ps7 | high-mobility group high mobility group box 1, pseudogene 7 | 180.15 | 0.048774 |

*** Fold change expressed as caffeine/vehicle.**

**Supplementary Table 2.** DAVID analysis identified significantly enriched pathways of DE genes in adult F1 mice treated with caffeine during E6.5-9.5.

| **Term** | **Count (% in pathway)** | **Genes** | **Fold enrichment** | **Corrected P-value** |
| --- | --- | --- | --- | --- |
| **GO:0006955~immune response** | 15 (15.46%) | H2-D1, H2-AB1, PSMB9, CFP, C1QA, GM8810, CD55, CXCL13, H2-EB1, CFH, H2-AA, TGTP2, GBP4, GBP3, GBP2 | 6.27 | 4.70E-05 |
| **GO:0009986~cell surface** | 11 (11.34%) | GM8810, RGMA, CD55, TNFRSF12A, APOE, CD274, H2-D1, CNTN2, H2-AA, H2-AB1, GPIHBP1 | 7.27 | 2.26E-04 |
| **GO:0005525~GTP binding** | 11 (11.34%) | BC023105, IGTP, GM12250, IRGM2, GM4841, IFI47, IIGP1, TGTP2, GBP4, GBP3, GBP2 | 6.16 | 0.002 |
| **GO:0003924~GTPase activity** | 7 (7.22%) | IGTP, IRGM2, IIGP1, TGTP2, GBP4, GBP3, GBP2 | 10.85 | 0.002 |
| **Signal peptide** | 32 (33.0%) | TNFRSF12A, IGFBP6, H2-D1, FSTL1, CFP, RGMA, GM8810, FOLR2, GRIN2C, APOE, CFH, SEMA3B, GPIHBP1, DNAJC1, THBS4, FGFBP3, LYZ2, KLK1B26, CPXM2, H2-AB1, C1QA, NPC1, CD55, PTGDS, CXCL13, CD274, H2-EB1, CNTN2, NPPB, H2-AA, BAMBI, GDF15 | 2.25 | 0.007 |
| **mmu05416:Viral myocarditis** | 6 (6.19%) | GM8810, CD55, H2-EB1, H2-D1, H2-AA, H2-AB1 | 10.77 | 0.010 |
| **GO:0019882~antigen processing and presentation** | 6 (6.19%) | GM8810, H2-EB1, H2-D1, H2-AA, H2-AB1, PSMB9 | 13.58 | 0.026 |
| **Propeptide:Removed in mature form** | 7 (7.22%) | RGMA, CD55, FOLR2, ACTA1, CNTN2, GBP2, PSMB9 | 6.62 | 0.036 |
